# Decline in Quinine

**Published:** 1889-03

**Authors:** 


DECLINE IN QUININE.—A strong demonstration of the hardship and injustice of the high tariff system that is in force in this country is furnished by the results which have followed upon the removal of the tariff upon quinine, which was brought about a few years ago by the efforts of medical men. This was done largely through the medical journals and by systematic appeals to congressmen throughout the country. The argument which had most weight was that the high price of quinine which then prevailed bore with great severity upon the poor, who were compelled to use large quantities of this drug on cacount of malarial sickness, which prevailed throughout all sections of the country. We well remember that the protectionists, and especially the proprietors of the large drug establishments who were engaged in the manufacture of the article protested against the removal of the tariff, claiming that it would not reduce the price in any material degree, but would probably increase it, and that, at all events it would be destructive of the interests of our citizens who were engaged in the manufacture of the article. But only a few years have past and these predictions of the manufacturers have all come to naught, and the price of quinine as sold by importers and manufacturers in large lots is now only 38 @ 45¢ per ounce, whereas it formerly rated at $2 50 to $2.75.

These figures speak for themselves and furnish a commentary upon the injustice of the tariff system more convincing and forcible than any mere argument in words, or any theories that the protectionists are able to present in favor of the protective tariff system.

